# Failure behavior of sandwich honeycomb composite beam containing crack at the skin

**DOI:** 10.1371/journal.pone.0227895

**Published:** 2020-02-03

**Authors:** M. Y. Al-Fasih, A. B. H. Kueh, M. H. W. Ibrahim

**Affiliations:** 1 Jamilus Research Center, Faculty of Civil Engineering and Built Environment, Universiti Tun Hussein Onn Malaysia, Parit Raja, Johor, Malaysia; 2 Office of Education in Sana’a, Sana’a, Yemen; 3 Department of Civil Engineering, Faculty of Engineering, Universiti Malaysia Sarawak, Kota Samarahan, Sarawak, Malaysia; University of Louisville, UNITED STATES

## Abstract

Skin crack defects can develop in sandwich honeycomb composite structures during service life due to static and impact loads. In this study, the fracture behavior of sandwich honeycomb composite (SHC) beams containing crack at the skin was investigated experimentally and numerically under four-point loading. Three different arrangements of unidirectional (UD) carbon fiber composite and the triaxially woven (TW) fabric were considered for the skins. The presence of a 10 mm crack at mid-span of the top skin, mid-span of the bottom skin, and mid-way between load and support of the top skin, respectively, were considered. Failure load equations of the load initiating the skin crack extension were analytically derived and then numerically developed using the *J*-integral approach. The crack extension failure mode dominated all cracked specimens except those with low-stiffness skin which were controlled by the compressive skin debonding and core shear failures.

## Introduction

Composite sandwich structures are highly suitable for applications as structural components that require low-mass and flexibly, such as in aerospace, marine, and civil engineering fields [1]. The composite sandwich structure consists of two thin but stiff composite skins separated by a light-weight core [2]. The skins located at the top and bottom carry the flexural load and the inner core performs by transmitting shear stresses between the skins while keeping their distance approximately constant during deformation under transverse loading [3]. With the increasing use of composites sandwich structures in primary load-bearing structures, there is an appreciable rise in interest in the influence of defects on their mechanical properties. Defects can develop during the service life of composite sandwich structures by static, impact or fatigue loading [4]. The strength of the sandwich structure is a result of the combination of the properties of the face skins, core, and interface. Any damage accumulated in one or more of these base materials will have an overall designation effect on the properties of the sandwich structure [5]. The defects of sandwich structures as a result of fabrication, assembly or during service phase can be commonly found in the forms of skin core debonding, crack at the core, and crack at the skins. The damage propagation behaviors of the sandwich structure containing interfacial crack due to debonding or impact damage have been widely examined analytically and experimentally in previous studies. By means of analytical description, Triantafillou and Gibson [6] observed that the debonding of the sandwich beam occurred if the existing cracks at the interface between the face and the core were relatively large. Zenkert [7] predicted the fracture loads for foam core sandwich beams through simulated debonding under four-point bending using fracture toughness values of simple specimens. Mouritz and Thomson [8] found that the strength of a sandwich composite beam was affected by the interfacial cracks in the case of compression and shear loaded specimens, while the strength was not affected under the flexural loading case. Thomson *et al*. [9] noticed that the static shear strength and the fatigue resistance suddenly decreased for the sandwich composite beam containing interfacial crack between skin and core when the crack length exceeded 20–30 mm due to the changing of the failure mechanism from the glass fiber reinforced polymer (GFRP) skin wrinkling to the foam core shear cracking. Recently, Mansourinik and Taheri-Behrooz [10] investigated numerically and experimentally the loading capacity of sandwich beams with initial core-skin debonding under four-point bending test. The results revealed that the initial debonding at the tension side of the beam had a negligible effect on the loading capacity. However, it had a remarkable effect at the compression side. The fracture toughness value of the honeycomb sandwich panels can change due to different thicknesses of skin and core materials as investigated from the double cantilever beam test observation [11]. Shah and Tarfaoui [12] measured the strain energy release rate (SERR) of Mode I and II for the face-plate foam-core interface using the double cantilever beam method, and the results displayed that the SERR values changed with the thickness of the core. Maleki and Toygar [13] had carried out analytical and experimental methods to examine the influences of core thickness and core density on fracture behavior of the glass-epoxy woven sandwich composites at different temperatures using Single Cantilever Beam (SCB) approach detecting the increase in SERR with temperature in all samples. It was noticed that a significant increase was for those with high core density at any temperature, while the outcome was independent of core thickness. Balaban and Tee [14] evaluated SERR of sandwich composite beams using a three-end notched flexure test and observed that it raised with the core thickness and density. A new fracture mode, namely the initiation of interlaminar delamination, kinking into face-sheet, and propagation of interlaminar delamination (IKP) was observed by Pan *et al*. [15] for honeycomb sandwich panels containing embedded artificial pre-crack using modified double cantilever beam (DCB) test. Berggreen *et al*., [16] used the face tearing experimental test for foam-cored sandwich structures and noticed that the crack propagated in the interface closely below the face skin of low core densities specimens while for those with higher core density, the crack tended to propagate in the laminate itself with extensive fiber bridging. Caner and Bažant [17] found that the plastic zones were developed in the foam core close to the edges of the loading platen for specimens with notches under the top skin while the quasi-brittle fracture was distributed in the foam core under tension for specimens with notches just above the bottom skin.

Having presented some works on the fracture behavior of sandwich structures, it is noted that a limited amount of research has been done to study their performance when containing crack in the core such as [18], in which the fracture load and the critical crack length were predicted through the stress intensity factors at the crack tips using the finite element (FE) method. Recently, Funari *et al*. [19] have attempted to analytically describe the macrocracks evolution in the core of sandwich structures through a moving mesh approach. In addition, Zenkert *et al*. [20] found that the blunt or sharp impact damage of the top skin of the composite sandwich panels decreased the compressive load carrying capacity of the composite skin. Although this reduction in performance is obviously significant, the influence of the face skin damage on the mechanical behavior of the sandwich structure has not been properly addressed. Therefore, the aim of this study is to investigate the effects of pre-existing static or impact damage on the flexural behavior of SHC beams and then to evaluate the failure load due to this damage using the numerical approach. Such a study would greatly help in the analysis and design of the composite sandwich structure containing skin defects as stability precaution can be then implemented to prevent any anticipated catastrophic failure.

## Experimental study

### Description of material and specimens

**Fig 1:**
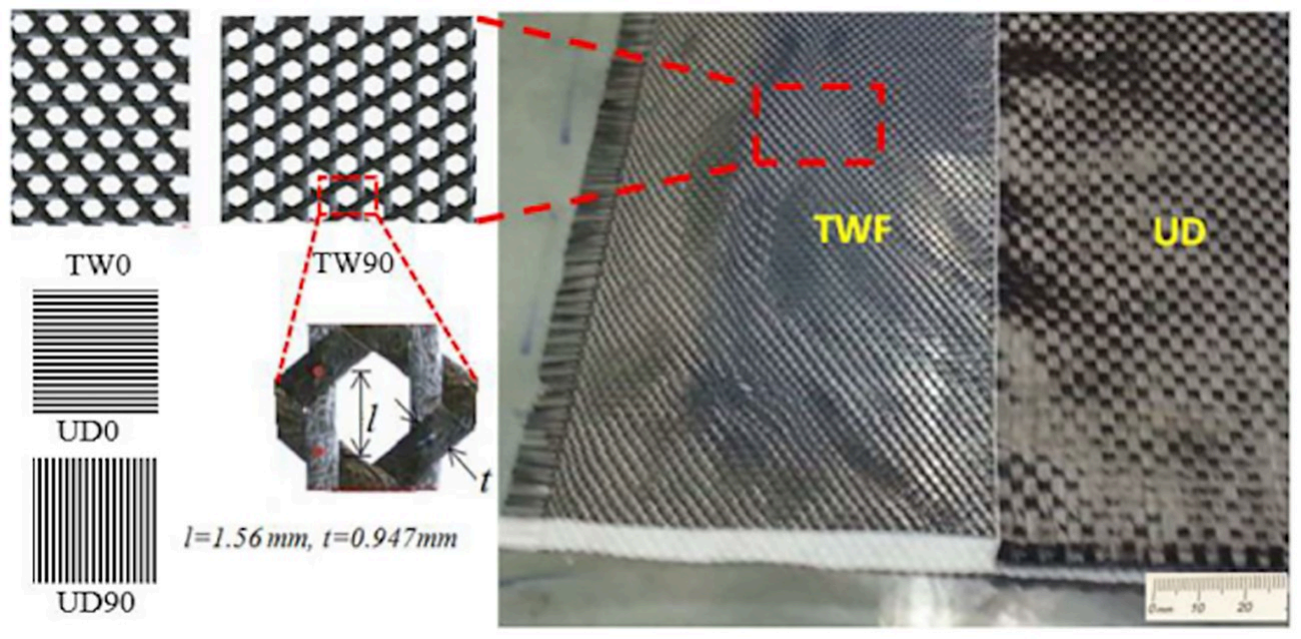
Dry TW fabric with its highlighted unit cell and dry UD carbon fiber sheets.

Specimens for the experimental study were constructed in the form of the SHC beam consisted of two CFRP face skins and a honeycomb core type of 5052 aluminum alloy. Two material types of CFRP were considered for the skins: Stitched unidirectional (UD) carbon fiber composite T350 supplied by Mapei, China, with a dry density of 300 gm/m^2^ and the triaxially woven (TW) fabric in ‘basic weave’ pattern from the Sakase Adtech, Japan, consisting of 1000-filament in each T300 carbon fiber tow (Toray Industries Inc., Japan), which has a dry density of 100 gm/m^2^ (see Fig 1).

**Fig 2:**
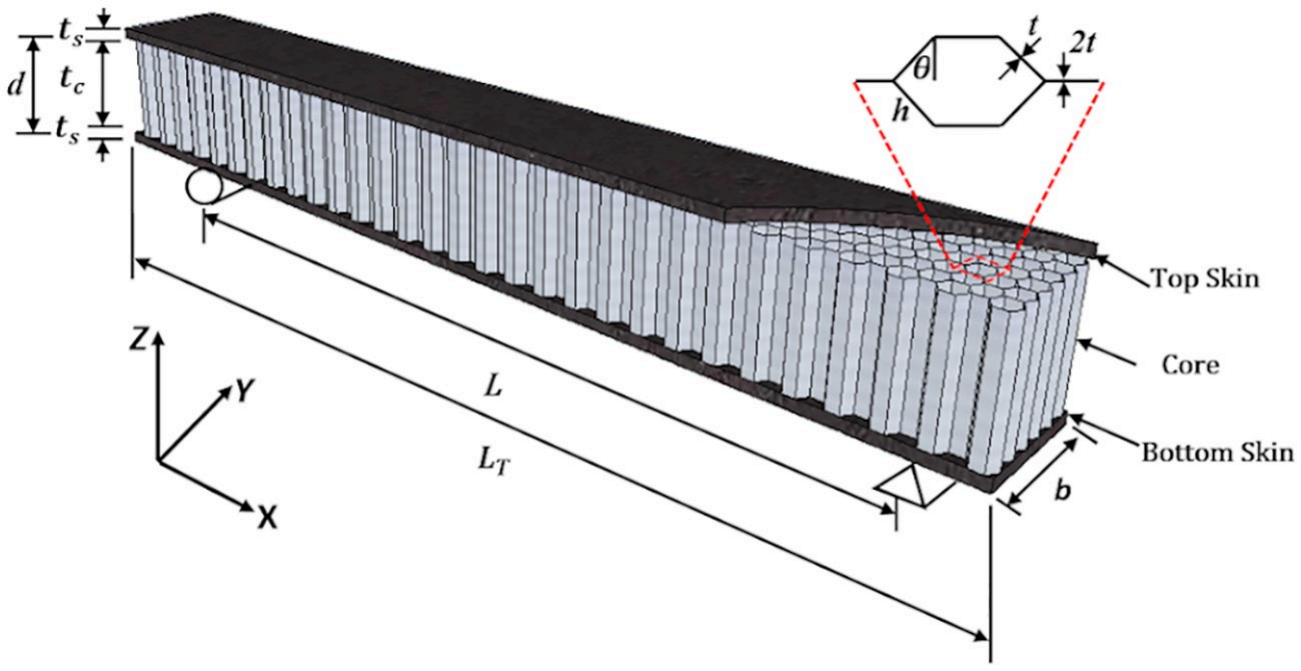
Schematic and the geometrical descriptions of the SHC beam depicting also the honeycomb core unit cell.

The commercial epoxy resin, Epicote 1006, and hardener from S&N Chemicals Sdn. Bhd, with the 5:3 proportions, was employed as the epoxy matrix. The geometrical descriptions of the beam and the hexagonal unit cell of the aluminum honeycomb core are shown in Fig 2 and numerically summarized in Table 1.

**Table 1:** Dimensions of the SHC beam and its aluminum honeycomb unit cell.

Total length of beam, *L*_*T*_ (mm)	350
Width of beam, *b* (mm)	30
Length of inclined wall, *h* (mm)	3.536
cell size, *l* (mm)	3.0
Thickness of the inclined wall, *t* (mm)	0.064
Cell wall angle, *θ* (°)	45

The CFRP skins were manufactured by means of the vacuum bagging method with three different stacking sequences; UD0/90 carbon fiber composite, which was made of plies of stitched UD with the stacking sequence of [0/90] with the nominal thickness of 0.8 mm and a density of 1000 g/m^2^; TW0/UD0 carbon fiber composite, which was made up of 0°-direction single ply TW and 0°-direction single ply UD with a nominal thickness of 0.6 mm and a density of 611 g/m^2^; and TW0/UD90 carbon fiber composite, which was made of 0°-direction single ply TW and 90°-direction single ply UD with a nominal thickness of 0.6 mm and a density of 611 g/m^2^. The SHC beam with UD0/90 skins has an overall density of around 225 kg/m^3^ while those with TW0/UD0 and TW0/UD90 skins have an overall density of around 184 kg/m^3^.

### The manufacturing process of specimens

**Fig 3:**
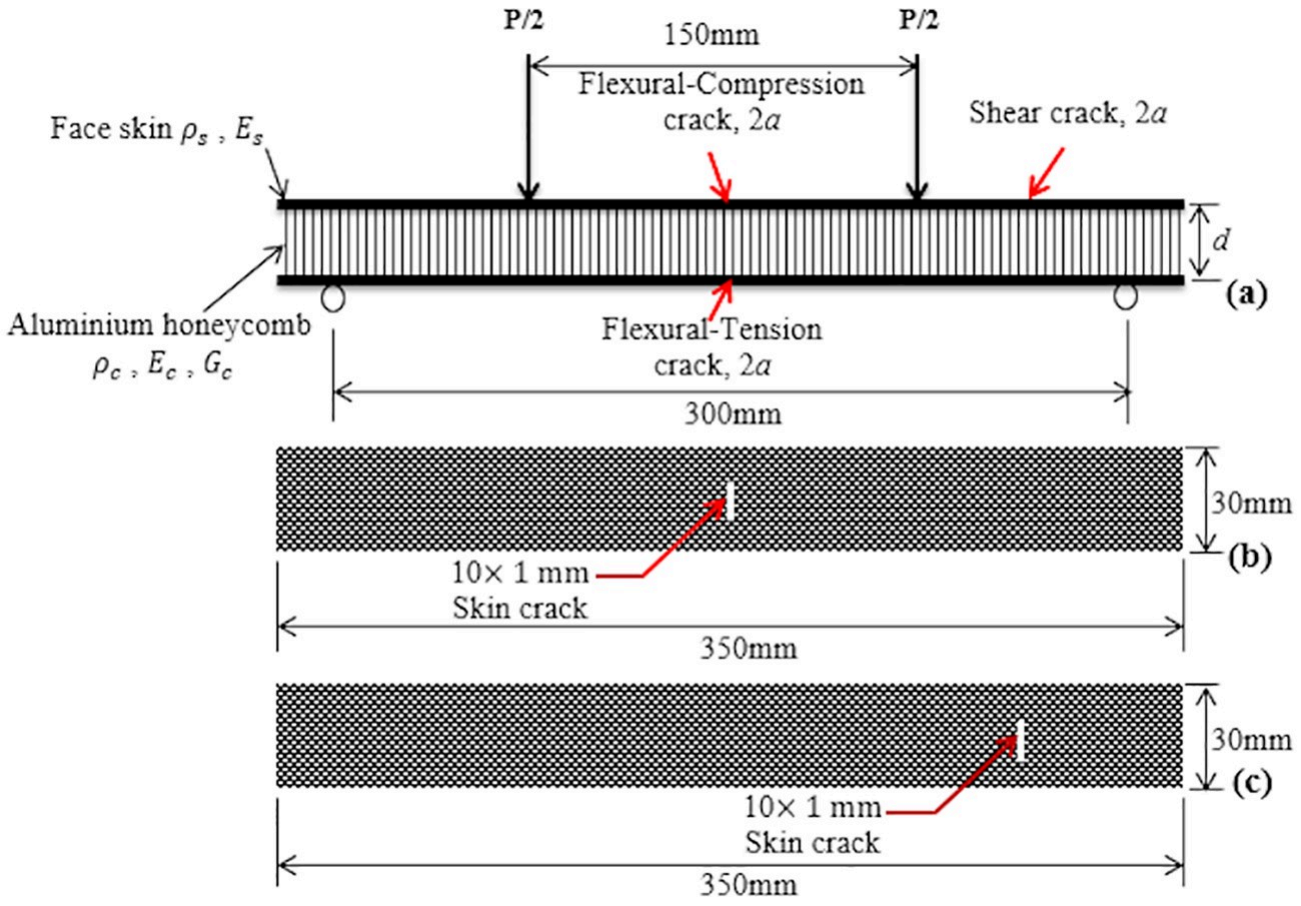
a) Lateral view of the four-point loading test, b) Top view of the skin showing crack located at the mid-span between the loading points and c) Top view of the skin showing crack located at the middle of the shear span.

The composite skins and the aluminum honeycomb core were designed according to the dimensions as presented in Table 2. To investigate the influence of the crack at the skin on the flexural behavior of the SHC beam using the four-point bending test, three cases were considered, i.e., flexural-compression, flexural-tension, and shear properties in flexure. For the case of the flexural-compression, the crack was positioned transversely at the mid-span of the top skin between the load points while it was positioned at the mid-span of the bottom skin between the load points for the case of flexural-tension (see Fig 3). On the other hand, for the case of the shear properties in flexure, the skin crack was positioned mid-way between one of the loading lines and support points as shown in Fig 3. In this position, the region of the beam containing the crack is subjected to shear loading. The skin crack was carefully introduced at the skins with a size of 10×1 mm using the Top Well cutting machine.

**Table 2:** Details of SHC beam specimens for a four-point loading flexural test.

Specimen name	Materials stacking sequence	*b* (mm)	*t*_*c*_	*L* (mm)	*L*_*T*_ (mm)
UD0/90	[UD0/UD90/core/UD90/UD0]	30	20	300	350
TW0/UD0	[TW0/UD0/core/UD0/TW0]	30	20	300	350
TW0/UD90	[TW0/UD90/core/UD90/TW0]	30	20	300	350

Three samples for each UD0/90, TW0/UD0, and TW0/UD90 SHC beam were prepared for flexural tests in the cases of crack-free and crack-containing specimens, respectively, according to the layers staking sequences as shown in Table 2. In the assembling process of the SHC beam, an equal distribution of the bonding resin (5:3 Epicote1006 to hardener) was applied on the skin-to-core interfaces, and then the aluminum honeycomb core was sandwiched immediately between the two skins. The vacuum bagging method was employed at a pressure of 0.0827 MPa for the complete sandwich structure lay-up for a period of 4 h to eliminate air. The composite was then cured at the room temperature of 25°C for 24 h.

For strain measurement, two strain gages were attached longitudinally 10 mm from the crack at the mid-span of the top most and bottom most laminate skin surfaces. Note that for the specimen’s nomination, TC, BC, and SC are added to the specimen codes at the end to represent the flexural-compression, flexural-tension, and shear cases, respectively. For example, UD0/90-BC denotes a sandwich beam with UD0/90 laminate skins tested under the flexural-tension load case.

### Test set-up and procedure

**Fig 4:**
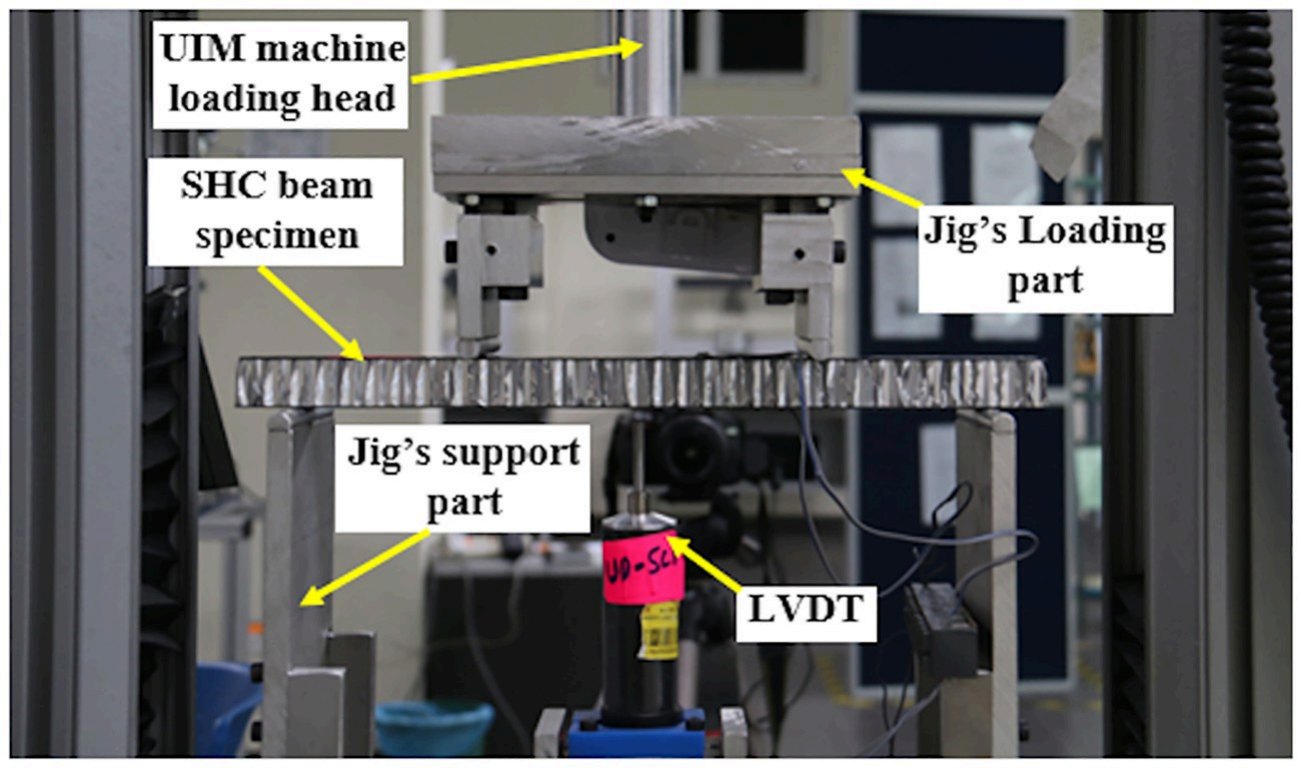
Actual test set-up for SHC beam specimen.

The static flexural test of the SHC beam was performed in accordance with the ASTM C393-02 [21]. Fig 4 shows the actual test set-up and instrumentation for the static flexural test of the SHC beam. The flexural tests were carried out using the Universal Instron machine 5567 with 30 kN load cell with a loading rate of 3 mm/min. Linear variable displacement transducer (LVDT) was placed in the middle part of the fixture to evaluate the mid-span displacement. The LVDT and uni-axial strain gages were connected to the data logger to evaluate the mid-span displacement and the longitudinal strain, respectively, during loading until final failure. Before each test, the loading pins were set to almost touching the top surface of the SHC beam specimen and the LVDT was set to touch the bottom surface of the SHC beam at the mid-span. The applied load, displacement, and strains were recorded. The test was stopped after the failure of the SHC beams was observed. The flexural stress, *σ*, under four-point bending for each applied load, which carried by the surface fibers of the top and bottom skins of the SHC beam, was calculated using the following expression [22]:

$$\sigma =\frac{PL(t_{s}+0.5t_{c})}{4bt_{s}t_{c}^{2}}$$ (1)

where *P* is the applied load, *L* is the supporting span, *t*_*s*_ is the skin thickness, *t*_*c*_ is core thickness and *b* is the beam width.

## Strain energy release rate and failure load analysis

The load, *P*, which leads to propagating the skin crack, can be determined by evaluating the SERR of the SHC beam following the analytical description of Triantafillou and Gibson [6], in which the failure load of debonding failure in the sandwich beam was determined in terms of the critical SERR. The SHC beam is loaded in four-point bending as shown in Fig 3. It has a length, *L*, a width, *b*, skin and core thicknesses, *t*_*s*_, and *t*_*c*_, respectively. The densities of the core and skin materials are *ρ*_*c*_, and *ρ*_*s*_ and Young’s modulus are *E*_*c*_, and *E*_*s*_, while the shear modulus of the core is *G*_*c*_.

The maximum deflection of the beam is the summation of the bending deflection, *δ*_*b*_, and shear deflection, *δ*_*s*_, of the beam. For a simply supported beam under four-point static bending with the loads at the location of *L*/4 and 3 *L*/4, the maximum deflection is given as [6]:

$$\delta =\frac{11PL^{3}}{768EI}+\frac{PL}{8A_{e}G_{c}}$$ (2)

where the flexural rigidity is $EI\approx \frac{E_{s}\;b\;t_{s}\;t_{c}^{2}}{2}$, and the shear rigidity is *A*_*e*_*G*_*c*_ ≈ *bt*_*c*_*G*_*c*_.

The SHC beam comprises a center skin crack of length, 2*a*, as shown in Fig 3. The load, *P*, which leads the skin crack to propagate, is found by determining the SERR of the SHC beam. The elastic strain energy in a perfect SHC beam is:

$$U=\frac{1}{2}\;P\;\delta$$ (3)

In the case of the SHC beams failed by the skin crack propagation, the elastic strain energy in the core is small compared with that in the skin. Then, the strain energy of the SHC beam is basically given by the strain energy of the skin. As the SHC beam loaded in four-point bending, the load is distributed uniformly over the length of the SHC beam. Thus, the energy per unit volume, *V*, is then determined by substituting for *δ*_*b*_ with Eq (3) as

$$\frac{U}{V}=\frac{1}{2}\;\frac{P\;\delta_{b}}{2\;b\;t_{s}\;L}=\frac{1}{2}\frac{11\;P\;(\;P\;L^{3})}{b\;t_{s}\;L\;(768\;E_{s\;}b\;t_{s}\;t_{c}^{2})}$$ (4)

Simplifying Eq (4) gives:

$$\frac{U}{V}=\frac{11}{1536}\;\frac{P^{2}L^{2}}{b^{2}\;t_{s}^{2}\;E_{s\;}t_{c}^{2}}$$ (5)

If the crack length, *a*, is equal to the skin width, *b*, the volume unloaded by a crack length, *a*, is *a*. *t*_*s*_ and the resultant release energy is:

$$U(a)=\frac{-\;{11\;P}^{2}L^{2}(a\;t_{s})}{1536\;b^{2}\;t_{s}^{2}{\;E}_{s}\;t_{c}^{2}}=\frac{-\;11\;P^{2}L^{2}a}{1536\;b^{2}t_{s}\;E_{s}\;t_{c}^{2}}$$ (6)

The Mode I SERR, *G*_*I*_, for in-plane tensile determination is:

$$\;G_{I}=-\frac{1}{b}\frac{\partial U}{\partial a}=\frac{11\;P^{2}L^{2}}{1536\;b^{3}t_{s}\;E_{s}\;t_{c}^{2}}$$ (7)

A fracture occurs when the Mode I SERR, *G*_*I*_, equals the critical SERR for the skin, *G*_*IC*_. Thus, this gives the failure load, *P*_*f*_ as:

$$P_{f}=\frac{11.82\;t_{c}b^{3/2}\;{\;(t_{s}\;E_{S}{\;G}_{IC}\;)}^{1/2}}{L}$$ (8)

The *J*-integral is another commonly used method for the analysis of cracks. It exceptionally describes the fracture behavior of elastic-plastic materials. The *J*-integral can be used for both elastic and plastic materials such as CFRP composites with a toughened matrix. The *J*-integral value is equal to the SERR, *G*, for the case of linear-elastic behavior [23]. An important advantage of the *J*-integral method is that it may also be applied for the analysis of cracks in elastic-plastic materials, provided no unloading occurs. The crack growth onset criterion for Mode I fracture using the critical *J*-integral, *J*_*IC*_, which is a material property, can be determined from the experiments. Therefore, Eq (8) can be written as:

$$\;P_{f}=\frac{11.82\;t_{c}b^{3/2}\;{\;(t_{s}\;E_{S}{\;J}_{IC}\;)}^{1/2}}{L}$$ (9)

This equation is valid when the assumption of the crack length, *a*, is equal to the skin width, *b*, thus the shape factor, $f(\frac{a}{b})$, as a function of crack length, *a*, and the skin width, *b*, is needed to determine the failure load of the SHC beam containing skin crack for *a* < *b*. For this purpose, Eq (9) should be written as:

$$P_{f}=f(\frac{a}{b})\frac{11.82\;t_{c}b^{3/2}\;{\;(t_{s}\;E_{S}{\;J}_{IC}\;)}^{1/2}}{L}$$ (10)

Thus, the determination of the shape factor $f(\frac{a}{b})$ will be the objective of the finite element (FE) modeling of the SHC beam containing the crack at the skin in the next section.

## Finite element modeling of composite sandwich structural behavior

**Fig 5:**
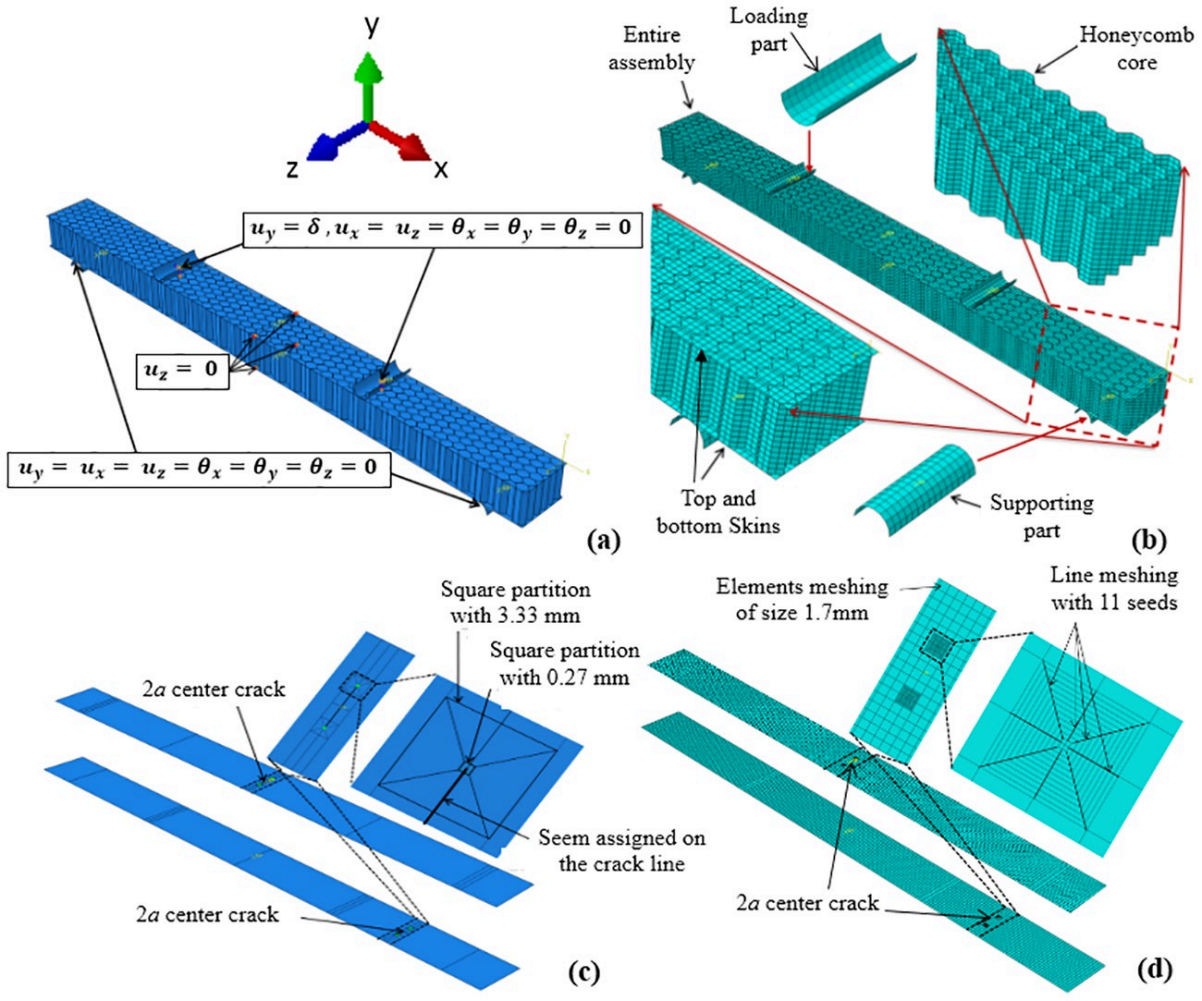
a) Numerical model of the SHC beam with boundary conditions, b) Numerical meshes for all parts of the SHC beam specimen, c) Geometry partitions for the skin containing 2*a* center crack at mid-span and shear locations, and d) Meshing of the skin containing 2*a* center crack at mid-span or shear locations.

The finite element (FE) modeling using the commercial software ABAQUS 6.13-1/standard [24] was developed to predict the behavior and the ultimate capacity of the SHC beams. The specimen and the loading set-up were simulated as identically as possible with the actual experimental conditions to have a reliable result. Four-point static bending behavior of the SHC beam was performed by developing a 3D finite-element model in the ABAQUS/standard domain. The boundary conditions of the model were applied as a constraint (see Fig 5a). The support and load spans were 300 mm and 150 mm, respectively. The skins were modeled as elastic materials, defined with properties established from the coupon tests as given in Table 3. The aluminum honeycomb core was modeled as an elastic-plastic material following the work of Ivañez and Sanchez-Seaz [25]. The Young’s modulus and Poisson’s ratio of the aluminum foil were 69000 MPa and 0.33, respectively.

**Table 3:** Mechanical properties of UD T350/EP-6001 and TW T300/EP-6001 composite.

Property	UD	TWF
Density, *ρ* (kg/m3)	998	221.5
Longitudinal stiffness, *E*_1_ (MPa)	123387	13126
Transverse stiffness, *E*_2_ (MPa)	8372	7608
Poisson’s ratio, *v*_12_	0.319	0.32
In-plane shear modulus, *G*_12_ (MPa)	4278	2798
Out-of-plane shear modulus, *G*_13_ (MPa)	4278	2798
Out-of-plane shear modulus, *G*_23_ (MPa)	2968	2859
Longitudinal tensile strength, *X*_*t*_ (MPa)	926	143
Longitudinal compressive strength, *X*_*c*_ (MPa)	345	41.5
Transverse tensile strength, *Y*_*t*_ (MPa)	8	65
Transverse compressive strength, *Y*_*c*_ (MPa)	57	5.2
Longitudinal shear strength, *S*_*t*_ (MPa)	19.45	11.5
Transverse shear strength, *S*_*c*_ (MPa)	19.45	11.5

The surface-based tie constraint was adopted to define the adhesive bonding between both skins and core to simulate a perfect bonding between them. Surface-to-surface contact was defined with the friction coefficient of 0.1 between the outer surface of both supporting and loading parts with the surfaces of skins at regions of contact only. As the friction occurs in the areas of skins that contacted the loading and the supporting parts, the friction coefficient was applied at the contact regions only where there is no friction in the remaining areas of the skins. Each contact region should not be less than the area that equal to the diameter of the supporting or loading part multiplied by the width of the skin.

Three types of models were developed to represent UD0/90, TW0/UD0, and TW0/UD90 SHC beams. The top and bottom skins, as well as the core, were meshed using 4-node shell element (S4) without the reduction integration. The UD0/90 CFRP skins were modeled to have a shell-composite section with a stacking sequence of [0/90] with a 0.4 mm thickness for each layer. The TW0/UD0 CFRP skins were modeled to have a shell-composite section with 0-direction for both TW and UD materials with thicknesses of 0.167 mm and 0.4 mm, respectively, while shell-composite section with 0°-direction and 90°-direction material orientations were considered for TW and UD materials, respectively, to represent the modeling of the TW0/UD90 CFRP skins. The honeycomb core foils were modeled to have a shell-homogenous section with a shell thickness of 0.1 mm. The supporting and loading parts (10 mm diameter) were discretized with the discrete rigid element (R3D4) (see Fig 5b). The refinements of the mesh were carried out until the optimal convergence plot was achieved. An average element size of 1.7 mm for the skins, 2.0 mm for supporting and loading parts, and 1.5 mm for the shell elements of aluminum honeycomb were considered with the total number of 38,416 elements for the finalized models.

The contour integral approach based on the FE method was used to evaluate the fracture mechanical parameter (*J*-integral) for the specimens containing 10 mm crack at the skin. This method requires the conforming of the mesh to the cracked geometry to explicitly define the crack front and to specify the virtual crack extension direction. Detailed meshes are generally required. The contour integral crack type was assigned to the squares around each crack tip using the normal crack plane method to specify the crack extension direction with the singularity of the mid-side node parameter of 0.25 (see Fig 5c and 5d). This definition moves the mid-side nodes on the element sides adjoining the collapsed edge to the 1/4 points of the elements [26]. At the crack tip, the element sides were collapsed with single-node-type degenerate element control.

The analysis was conducted using the geometrically nonlinear static step. The failure of the SHC beam models was defined as when the maximum strength in the elements was exceeded either in terms of the maximum compressive, tensile or shear strength of the material. The deflection and bending stress-strain relationships at the top-most and bottom-most shell elements at the mid-span results were plotted and then compared with those of the experimental for the SHC beams containing crack at the skin for verification purpose.

## Shape factor, $f(\frac{a}{b})$, determination

The modeling of the TW0/UD0 type SHC beam was considered to determine the shape factor, $f(\frac{a}{b})$, for determining the failure load of the SHC beam containing skin crack when *a* < *b* as discussed in Eq (10). Seven different crack length to beam’s width ratios of $\frac{a}{b}=0.22$, 0.33, 0.44, 0.55, 0.66, 0.77, and 0.88 were modeled for each of the TW0/UD0-BC, TW0/UD0-TC, and TW0/UD0-SC SHC beams. The same modeling procedure as that described for the 10 mm crack length was considered. The reaction force, which equals the applied load, and the *J*-integral values were determined from the ABAQUS. From the applied load-time and *J*-integral-time curves, the highest *J*-integral value for any contour at a given load, *P*, was chosen and considered as the best approximation to the real far-field *J*. Then, the relationships of $f(\frac{a}{b})=\frac{PL}{11.82\;t_{c}b^{3/2}\;{\;(t_{s}\;E_{S}\;J_{I}\;)}^{1/2}}$ versus $\frac{a}{b}$ were plotted, such that the shape factors for each crack location were determined using the polynomial fitting.

## Results and discussion

### Load-deflection behavior

**Fig 6:**
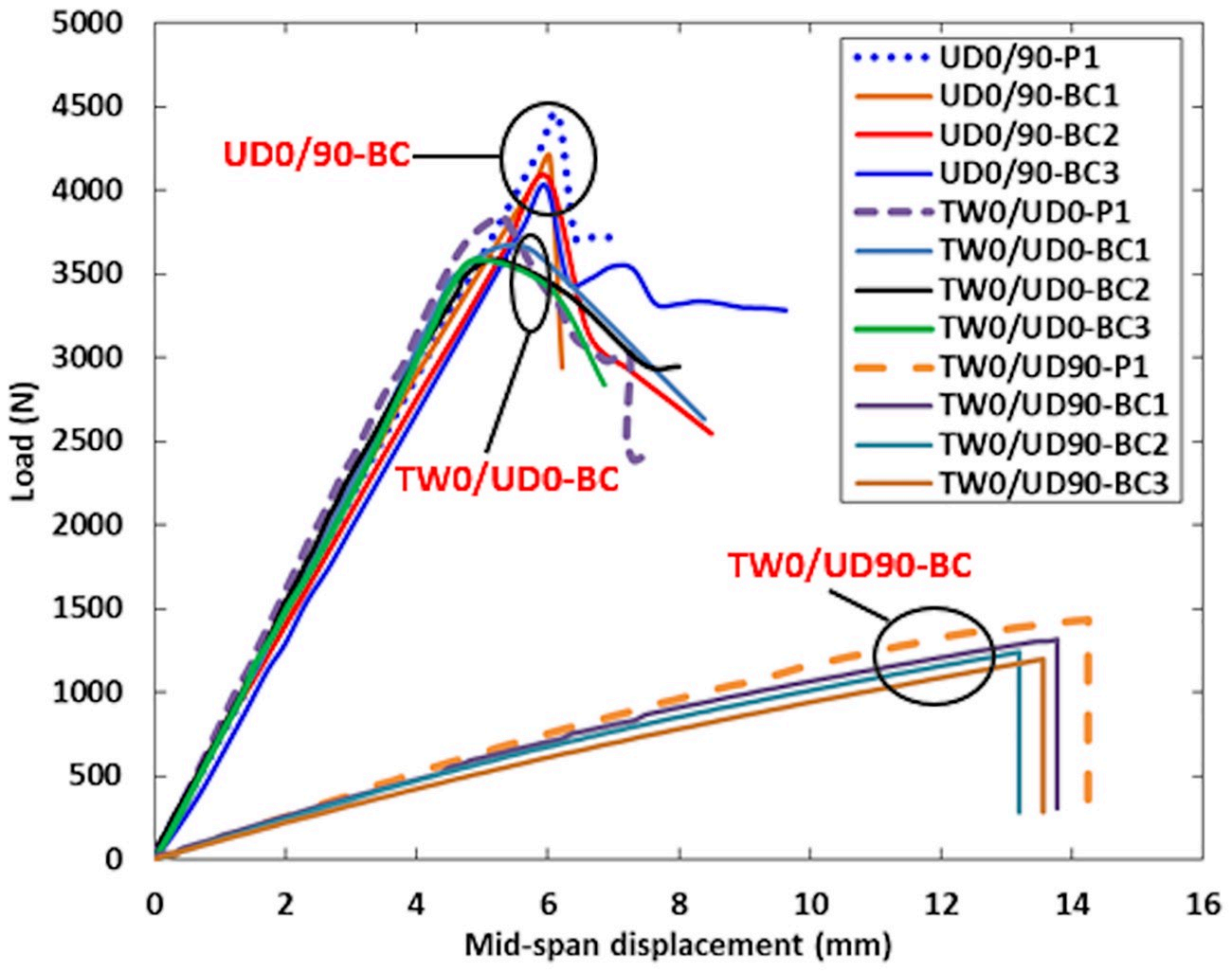
Load against mid-span displacement curve for crack-free SHC beams and those containing pre-existing 10 mm transverse skin crack at the tension side.

**Fig 7:**
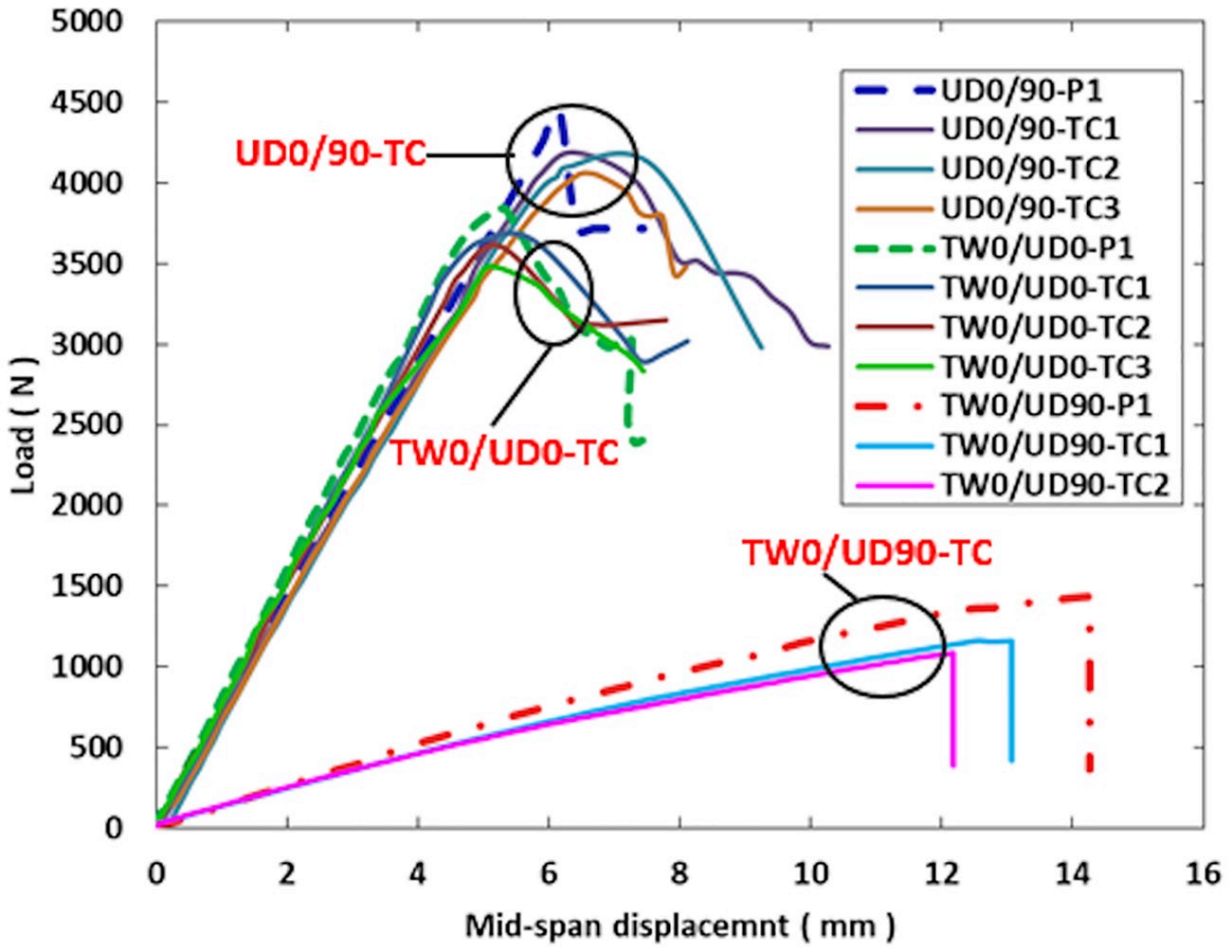
Load against mid-span displacement curve for crack-free SHC beams and those containing pre-existing 10 mm transverse skin crack at the compression side.

**Fig 8:**
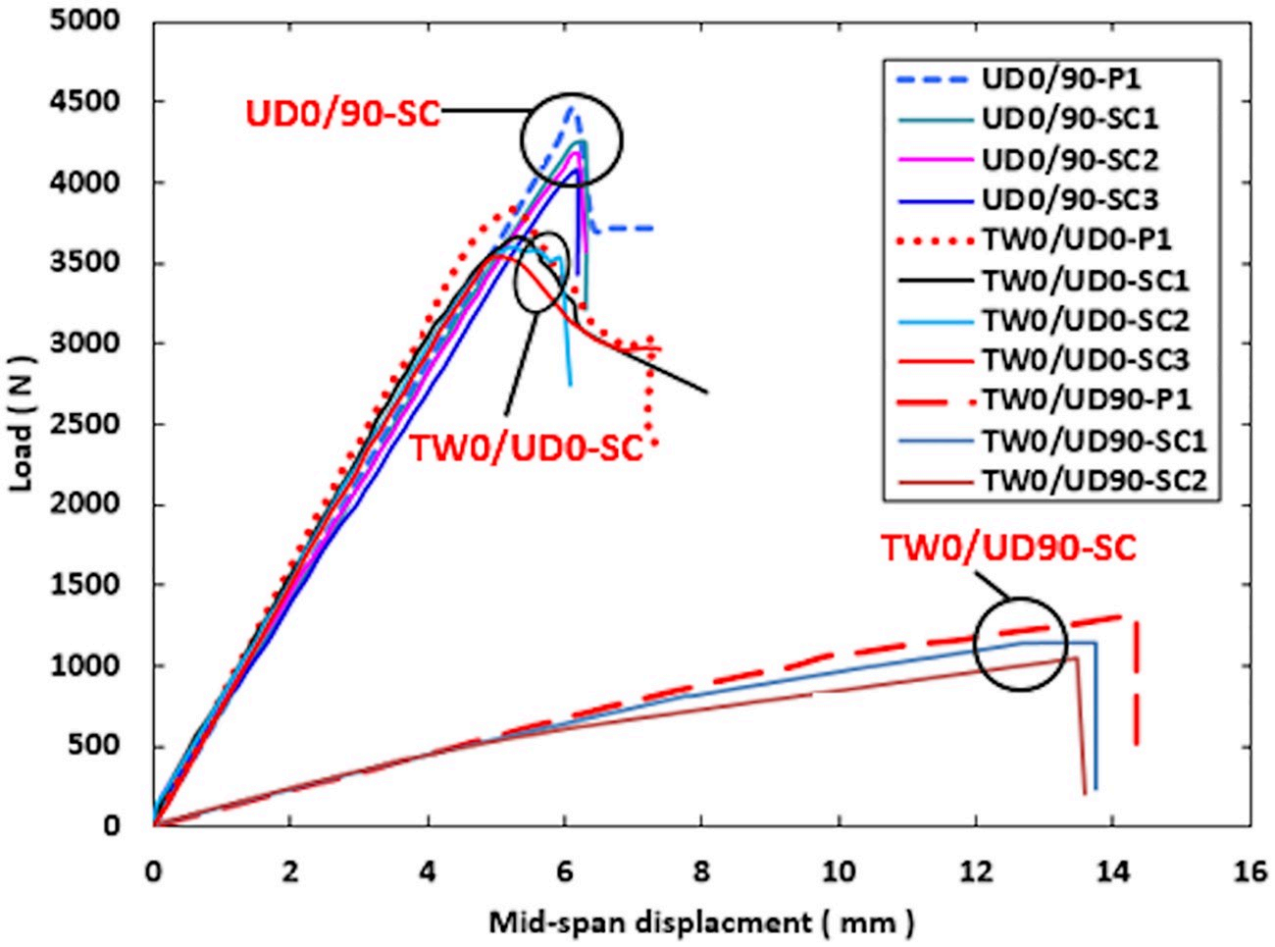
Load against mid-span displacement curve for crack-free SHC beams and those containing pre-existing 10 mm transverse top skin crack at shear span.

Figs 6–8 show the load against mid-span displacement curves of the SHC beams containing 10 mm crack length at the skin as compared with those crack-free under four-point bending. In the plots, P1 represents the crack-free control case. The load-displacement curves of both crack-free SHC beams and those containing skin crack for UD0/90 and TW0/UD0 specimens increased linearly until the maximum failure load. Meanwhile, the TW0/UD90 SHC beam specimens displayed a linear behavior up to a deflection of 6 mm, Then, the curves extend with a small nonlinearity due to stiffness softening until failure. Sudden failure was observed for UD0/90-BC, UD0/90-SC, and all TW0/UD90 SHC beam specimens. The load-deflection curves dropped gradually for TW0/UD0-TC and UD0/90-TC SHC beam specimens. The average maximum failure load decreased to 6% for UD0/90 and TW0/UD0 SHC beam specimens while it reduced to 15% for TW0/UD90 specimens due to the presence of 10 mm face skin crack compared with the crack-free SHC beam specimens. This indicates that the presence of a 10 mm face crack has a slight effect on the load-displacement curves of SHC beams in the cases of flexural-compression, flexural tension, or shear cracks.

### Stress-strain behavior of SHC beams containing face crack

The flexural stresses were carried by the surface skin fibers of SHC beam skins and determined according to Eq (1). The average results as summarized in Table 4 indicate that the strains at the mid-span of the cracked bottom or top skin of SHC beam specimens decreased by around 50% of those at the mid-span of the opposite, perfect skin of the same SHC beams specimen. This is due to the dissipation of the strains around the center of the crack concentration around the crack tips. Meanwhile, for the cracked specimens at the shear region of top skin, the strain at the mid-span of the top surface of SHC beams matches the strain at mid-span of the bottom. However, the fiber composite skins behaved slightly stiffer in compression than in tension. Note that no strain gages were attached close to the shear crack.

**Table 4:** Summary of important stress-strain parameters of SHC beams under four-point loading.

Property	Type	UD0/90	TW0/UD0	TW0/UD90
**Flexural stresses (MPa)**	P1	369	420	158
	BC	345	398	138
	TC	347	394	135
	SC	351	398	129
**Bottom-face strain (microstrain)**	P1	5263	4957	12798
	BC	1924	2531	6928
	TC	5026	4416	10946
	SC	5460	4518	13802
**Top-face strain (microstrain)**	P1	4880	4992	13060
	BC	4506	4382	13559
	TC	2117	1724	8516
	SC	5532	4567	13569

### Failure behavior

**Fig 9:**
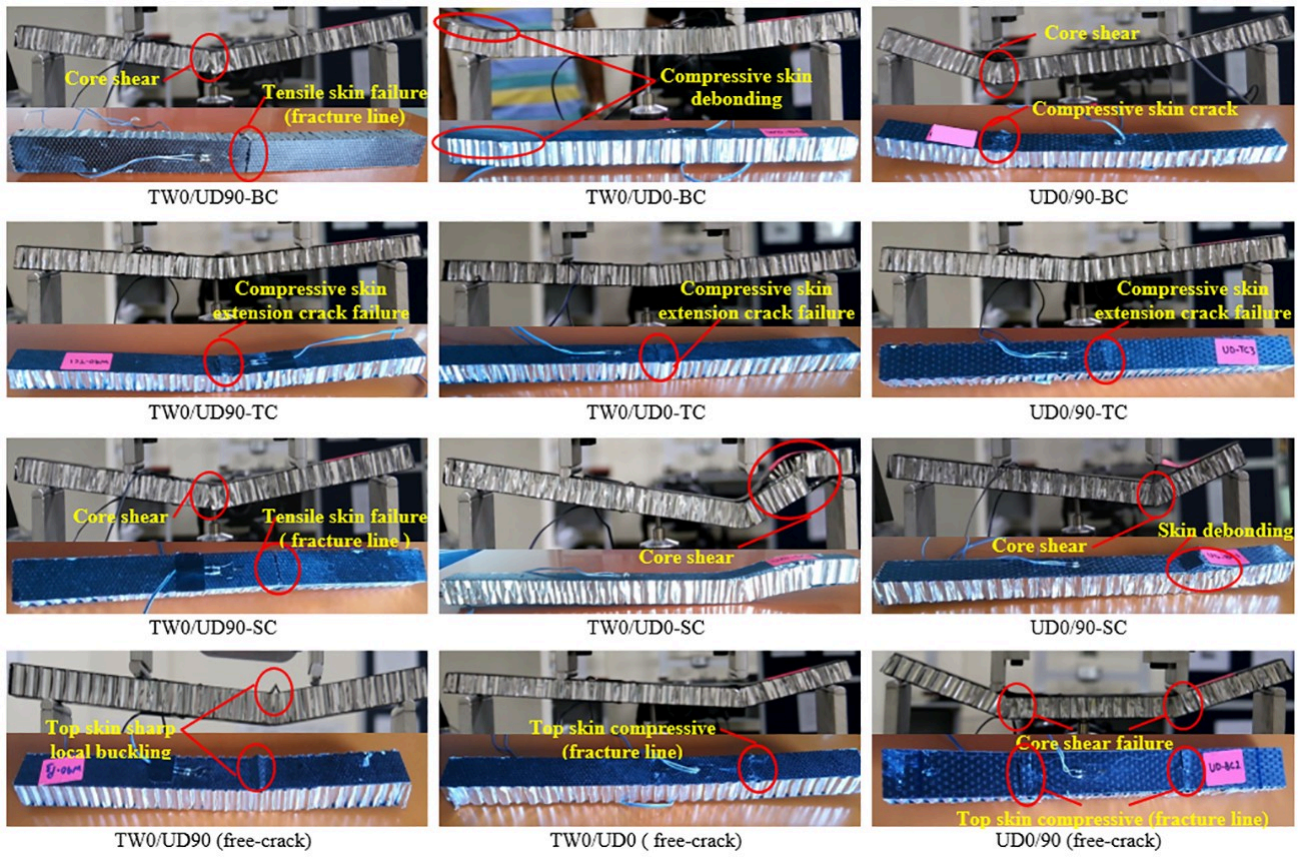
The typical failure mode of crack-free SHC beam specimens and those containing 10 mm skin crack.

Fig 9 shows the typical failure mode of the crack-free SHC beam specimens and those containing 10 mm skin crack. In Fig 9, the crack-free UD0/90 SHC beam specimens failed due to core shear failure followed by a compressive failure of the top compressed skin face underneath the loading points. It was noted that the crack-free TW0/UD0 SHC beam specimens failed due to compressive failure of the top face, but only underneath one point-load location while the crack-free TW0/UD90 SHC beam specimens failed due to local buckling of the compressive skin between the loading points. On the other hand, the experimental results of the specimens containing crack at the skins show that all flexural-compression SHC beam specimens failed in a brittle manner due to the extension of the crack at the compressive skins, followed directly by the specimen bending with the plastic hinge formation at the mid-span. Moreover, the extension crack failure controlled the flexural-tension SHC beam specimens with low stiffness skins, TW0/UD90, but not the specimens with stiff skins such as UD0/90 or TW0/UD0. TW0/UD90-BC failed due to the extension of crack at the tension skin, bent immediately with plastic hinge formation with core shear at mid-span. Meanwhile, the UD0/90-BC SHC beam failed due to core shear, followed by the compressive skins resulting fracture line beneath the loading point. TW0/UD0-BC SHC beam failed due to premature compressive skin debonding. These failure modes were observed in the crack-free UD0/90 and TW0/UD0 SHC beam specimens. This illustrates the small effect of pre-existing crack on the load-deflection curves behavior, which was readily discussed and shown in Figs 6–8. It is worth noting that TW0/UD90-SC SHC beam specimens failed due to the tensile skin failure by means of the fracture line parallel to the specimen width, although there was a pre-existed crack at the shear location in the top skin. This indicates that the tensile skin failure controls the failure of the TW0/UD90-SC SHC beam specimen more than the crack extension failure. For TW0/UD0-SC and UD0/90-SC SHC beam specimens, a close observation during the test informed that the crack initiated in the interfacial epoxy matrix near to the skin crack location, and upon further loading, it grew into the specimen resulting in a sudden compressive skin debonding before an immediate core shear occurred, at which point the SHC beam failed. Mouritz and Thomson [8] and Zenkert [7] also observed this type of shear failure in polymer foam sandwich composite containing interfacial cracks.

### Numerical results

**Fig 10:**
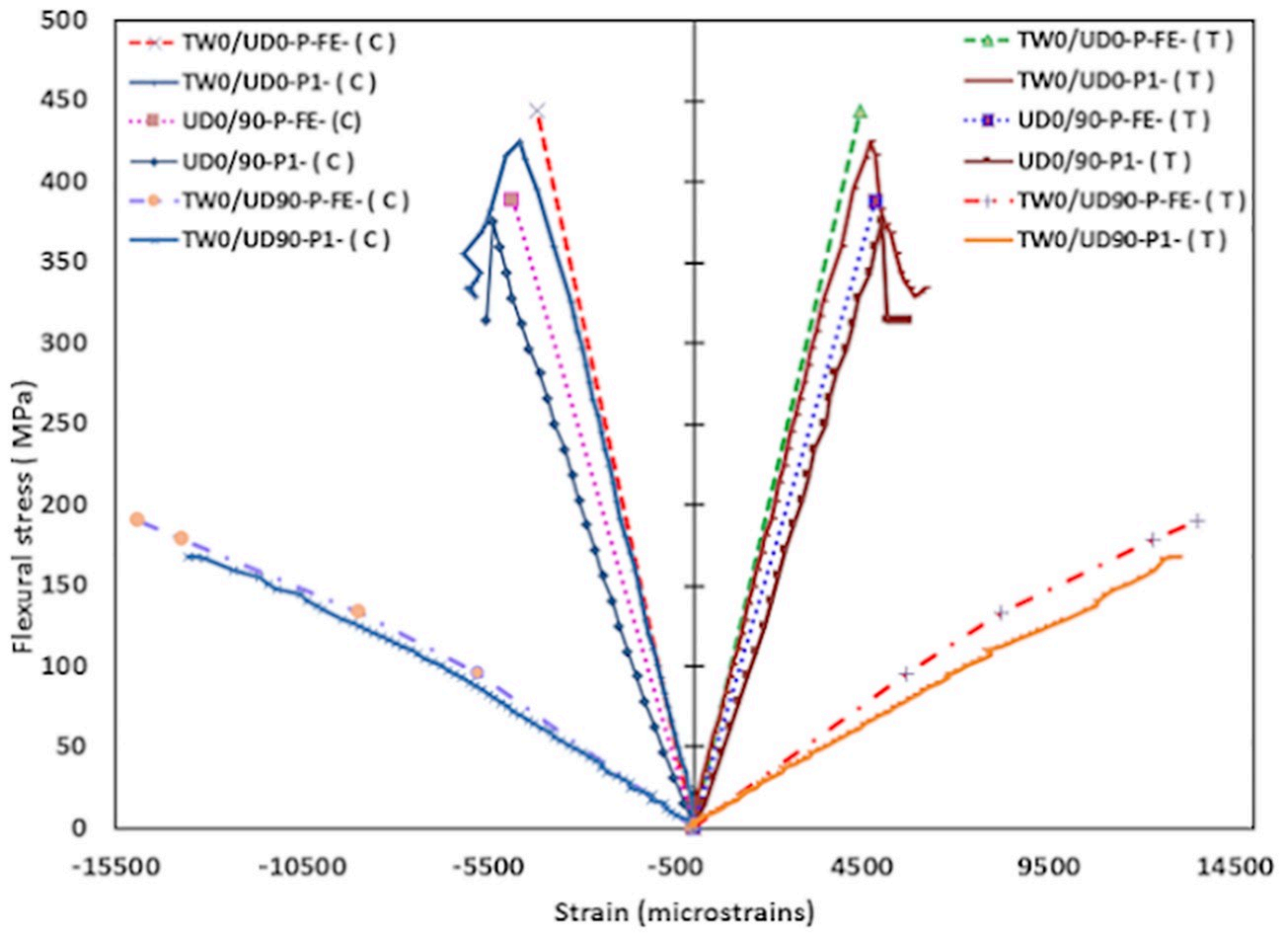
Flexural stress-strain behavior for SHC beams containing pre-existing 10 mm transverse skin crack at the compression side.

The FE model accurately captured the stress-strain behavior of the SHC beams as shown in Fig 10. In the plots, FE, (C), (T) added to the specimen codes at the end to represent the FE model, compressive stress-strain and tensile stress-strain curves, respectively, for each specimen type. A good agreement of the experimental result with the FE numerical predicted stress-strain relation was observed. In Fig 10, the stress-strain curves for all UD0/90 and TW0/UD0 SHC beam specimens increased linearly before any failure, while a slight non-linear behavior in stiffness was started at around 50% of the maximum failure stress for all TW0/UD90 SHC beam specimens. The differences between the average experimental and FE results in terms of the stiffness for all types of skins are less than 25%. These differences may be attributed to the fabricating process that produced some variability in the dimensions of both aluminum honeycomb core and CFRP facings.

**Fig 11:**
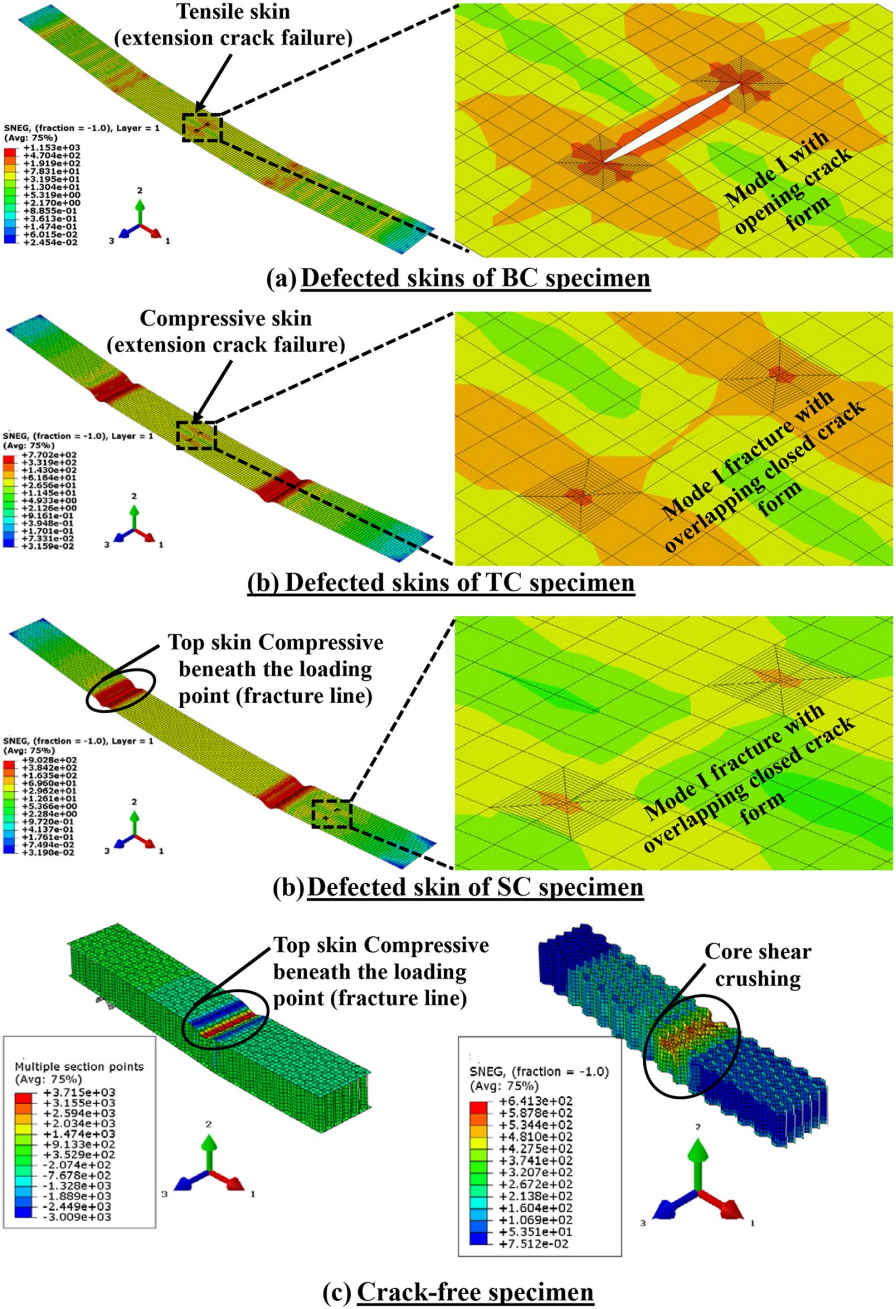
Oblique views of the deformed shape and stress distribution by FE modeling which indicating the failure modes of the specimens.

Fig 11 shows the skin deformation and stress distribution generated which indicating the failure modes using the FE modeling for defected skins of BC, TC, SC, and crack-free specimens, respectively. The enlarged views of the crack zones are shown at the right side of each deformed skin to illustrate the direction of the crack opening deformation and the stress distribution contour around the crack tips. As can be seen in the enlarged figures, the crack of the BC type open in the 1-direction, in which the *J*-integral implies an almost pure Mode I fracture while the cracks of TC and SC model types are completely closed with overlapping displacement in the 1-direction, which indicates that the *J*-integrals are of Mode I but in an opposite manner. In the enlarged figures, the stress and strain are also concentrated around the crack tips and they are dissipated around the center of the crack. This is confirming that the strain at mid-span of the cracked face skin is 50% lower than the strain at the mid-span of the opposite, perfect skin of the same SHC beams specimen.

**Fig 12:**
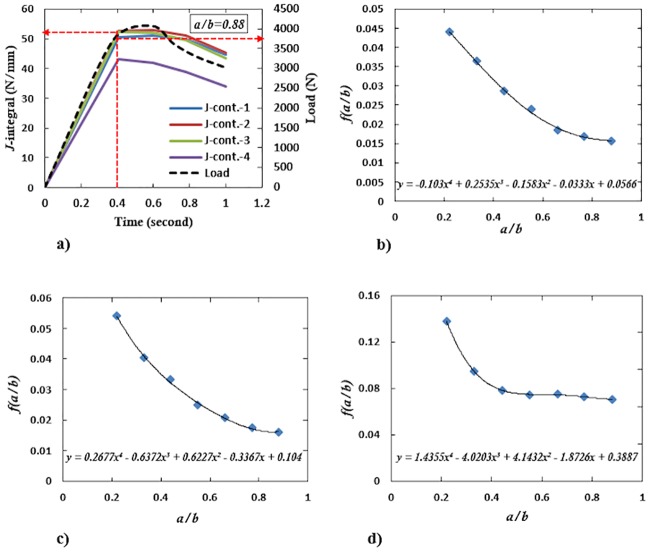
a) *J*-integral and load versus time curves for $\frac{a}{b}=0.88$ of TW0/UD0-BC SHC beam with pre-existing skin crack and, the corresponding $f(\frac{a}{b})$ versus *a*/*b* of the b) TW0/UD0-BC, c) TW0/UD0-TC and d) TW0/UD0-SC SHC beam.

In general, good agreement in numerical and experimental methods was observed in terms of stress-strain curves, stress, and strain distribution contours, as well as failure modes. Having verified the capability of the numerical technique, we next proceed to explore mainly the *J*-integral curve behavior of the SHC beams containing crack at the skin. The load-time and the *J*-integral-time curves for $\frac{a}{b}=0.22$, 0.33, 0.44, 0.55, 0.66, 0.77 and 0.88 of TW0/UD0-BC, TW0/UD0-TC and TW0/UD0-SC SHC beams with pre-existing skin crack was plotted. Fig 12a shows an example for the load-time and the *J*-integral-time curve of $\frac{a}{b}=0.88$. In Fig 12a, the legends J-cont.1 to J-cont.4 indicate the various contour numbers considered. The *J*-integral was evaluated by ABAQUS 6.13 using the finite deformation option, NLGEOM. The plots show that all *J*-integral values are positive and consistent except for the curve denoted J-cont.4 with a small dispersion, which implies that it is close to the boundary of the specimen. The highest value of *J*-integral from any contour with its corresponding load value (at the same time step) are the best values chosen to be defined as *J*_*IC*_ for each $\frac{a}{b}$ ratio as shown in Fig 12a.

These values were substituted in the expression $f(\frac{a}{b})=\frac{Pl}{11.82\;t_{c}b^{3/2}\;{\;(t_{s}\;E_{S}{\;J}_{I}\;)}^{1/2}}$, from which the relationships of $f(\frac{a}{b})$ versus $\frac{a}{b}$ were then plotted for each specimen types as shown in Fig 12b, 12c and 12d. The fitting of these curves that conform to Eq (10) can be written as follows:

$$P_{f}=f(\frac{a}{b})\frac{11.82\;t_{c}b^{3/2}\;{\;(t_{s}\;E_{S}{\;J}_{Ic}\;)}^{1/2}}{L}$$ (11)

where

$f(\frac{a}{b})=-0.103{\left(\frac{a}{b}\right)}^{4}+{0.253(\frac{a}{b})}^{3}-{0.158(\frac{a}{b})}^{2}-0.0333(\frac{a}{b})+0.056$, for TW0/UD0-BC SHC beams.

The same procedure was followed for TW0/UD0-TC and TW0/UD0-SC SHC beam types to get the $f(\frac{a}{b})$ versus *a*/*b* curves as shown in Fig 12. The $f(\frac{a}{b})$ was determined as:

$$0.267{\left(\frac{a}{b}\right)}^{4}-{0.637(\frac{a}{b})}^{3}+{0.622(\frac{a}{b})}^{2}-0.336(\frac{a}{b})+0.104\;(\text{for}\;\text{TW}0/\text{UD}0-\text{TC}),$$

and

$$1.435{\left(\frac{a}{b}\right)}^{4}-{4.02(\frac{a}{b})}^{3}+{4.143(\frac{a}{b})}^{2}-1.872(\frac{a}{b})+0.388\;(\text{for}\;\text{TW}0/\text{UD}0-\text{SC})$$

According to the plots, it is apparent that the shape factors are tending to a constant for TW0/UD0-BC and TW0/UD0-TC SHC beam specimens when $\frac{a}{b}\geq 0.77$ but converges to a constant when $\frac{a}{b}\geq 0.55$ for TW0/UD0-SC SHC beam specimens.

## Conclusion

This paper investigated the effects of pre-existing static or impact damage on the flexural behavior of SHC beams under four-point loading. The UD0/90, TW0/UD0, and TW0/UD90 SHC beam specimens were investigated in the presence of pre-existing compression crack, TC, tension crack, BC, and shear crack, SC. The compression crack, TC, and tension crack, BC, were introduced transversely at the mid-span of the top and the bottom skins between the load points, respectively, while the shear crack, SC, at mid-way between one of the loading line and support point. The experimental results showed that the flexural-compression SHC beam specimen failed in a brittle manner in terms of compressive skin failure resulted from the extension of the compression crack. The extension crack failure controlled the flexural-tension SHC beam specimens with low-stiffness skins, TW0/UD90, but not the SHC beam specimen with stiff skin, such as UD0/90 or TW0/UD0 SHC beam specimens, in which they failed due to core shear, followed by the compressive skins resulting in fracture line beneath the loading point. The failure of the shear SHC beam specimens with stiff skins failure was initiated by the crack in the interfacial epoxy matrix near to the skin crack location, and upon further loading, grew into the specimen resulting in a sudden compressive skin debonding with subsequent immediate core shear, at which point the SHC beam failed. Specimens with lower stiffness skins, TW0/UD90 SHC beams, were not affected by the shear crack as they failed by a fracture in a line form parallel to the width direction at the tension skin. The stiffness and strength of the SHC beam slightly decreased to 6% for UD0/90 and TW0/UD0 SHC beam specimens while they reduced to15% for the TW0/UD0 SHC beam due to the presence of 10 mm skin crack length compared with the perfect SHC beam specimens.

The equation of failure load prediction of SHC beam that causes the skin crack extension was analytically derived and numerically developed for different crack lengths as in Eq (11). In general, the presented skin crack extension failure mode can be considered as a new failure mode compared with other existing failure modes such as skin wrinkling, compressive or tensile skin failure, and core shear. Therefore, the load failure prediction equation developed by the analysis and FE numerical approaches here can be used to determine whether the skin crack is the critical failure mode for a given beam geometry and initial crack length.
